# Sebetralstat for On‐Demand Treatment of Mucosal Hereditary Angioedema Attacks in KONFIDENT‐S

**DOI:** 10.1002/clt2.70118

**Published:** 2025-11-18

**Authors:** Jonathan A. Bernstein, Emel Aygören‐Pürsün, Mauro Cancian, Danny M. Cohn, Timothy Craig, Vesna Grivcheva‐Panovska, Anthony Jordan, William R. Lumry, Inmaculada Martinez‐Saguer, Isaac Melamed, Kazumasa Ohmura, Jonny Peter, Marc A. Riedl, Daniel F. Soteres, Petra Staubach, Marcin Stobiecki, Ya‐Hsiu Chuang, Michael D. Smith, Christopher M. Yea, Paul K. Audhya, Andrea Zanichelli, Henriette Farkas

**Affiliations:** ^1^ University of Cincinnati College of Medicine and Bernstein Clinical Research Center Cincinnati Ohio USA; ^2^ University Hospital Frankfurt Goethe University Frankfurt Frankfurt Germany; ^3^ Departmental Allergy Division Department of Systems Medicine University of Padua Padua Italy; ^4^ Amsterdam University Medical Center University of Amsterdam Amsterdam the Netherlands; ^5^ Department of Medicine Pediatrics MFM, and Biomedical Sciences Penn State University Hershey Pennsylvania USA; ^6^ Vinmec‐VinUni Institute of Immunology Vin University Hanoi Vietnam; ^7^ Center of Allergy and Clinical Immunology, Vinmec Times City Hospital Hanoi Vietnam; ^8^ University Clinic of Dermatology School of Medicine University Saints Cyril and Methodus Skopje North Macedonia; ^9^ Department of Clinical Immunology and Allergy Auckland City Hospital Te Toka Tumai, Auckland New Zealand; ^10^ AARA Research Center Dallas Texas USA; ^11^ HZRM Haemophilia Center Rhein Main Frankfurt Germany; ^12^ IMMUNOe Research Center Centennial Colorado USA; ^13^ Institute of Preventive Medical Sciences Health Sciences University of Hokkaido Hokkaido Japan; ^14^ Division of Allergology and Clinical Immunology Department of Medicine Groote Schuur Hospital University of Cape Town Cape Town South Africa; ^15^ Allergy and Immunology Unit University of Cape Town Lung Institute Cape Town South Africa; ^16^ University of California San Diego La Jolla, California USA; ^17^ Asthma & Allergy Associates PC Colorado Springs Colorado USA; ^18^ Department of Dermatology and Allergy University Medical Center Mainz Germany; ^19^ Department of Clinical and Environmental Allergology Jagiellonian University Medical College Krakow Poland; ^20^ KalVista Pharmaceuticals Framingham Massachusetts USA; ^21^ Operative Unit of Medicine Angioedema Center IRCCS Policlinico San Donato Milan Italy; ^22^ Dipartimento di Scienze Biomediche per la Salute University of Milan San Donato Milanese, Milan Italy; ^23^ Hungarian Angioedema Center of Reference and Excellence Department of Internal Medicine and Haematology Semmelweis University Budapest Hungary

**Keywords:** angioedema, HAE‐C1INH, hereditary angioedema, mucosal, on‐demand

## Abstract

**Background:**

Hereditary angioedema (HAE‐C1INH) attacks involving mucosal tissue may progress rapidly and often lead to substantial morbidity. Severe laryngeal attacks can be fatal without prompt administration of on‐demand treatment. This prespecified interim analysis evaluated the safety and effectiveness of sebetralstat in laryngeal and abdominal attacks in the ongoing, 2‐year, open‐label extension KONFIDENT‐S study (NCT05505916).

**Methods:**

Eligible participants ≥ 12 years with HAE‐C1INH self‐administered sebetralstat 600 mg film‐coated tablets with an optional second dose after 3 h, if warranted. Primary outcome: incidence of treatment‐emergent adverse events (TEAEs). Secondary outcomes: times to beginning of symptom relief, reduction in severity, and complete resolution.

**Results:**

At data cutoff (Sep 14, 2024), 32 laryngeal (16 participants) and 533 abdominal only attacks (102 participants) were treated with sebetralstat. Seven (43.8%) participants with laryngeal attacks experienced 14 TEAEs whereas 36 (35.3%) participants with abdominal attacks experienced 91 TEAEs. No difficulty swallowing sebetralstat was reported. Median (IQR) time to treatment: 11.5 min (1.0–34.0) and 20.0 min (1.0–61.0) for laryngeal and abdominal attacks, respectively; time to beginning of symptom relief: 1.29 h (0.76–3.02) and 1.27 h (0.76–3.54); reduction in attack severity: 4.25 h (1.22 to > 12) and 3.52 h (1.26 to > 12); complete attack resolution: 12.69 h (5.11 to > 24) and 15.17 h (4.46 to > 24). Most mucosal attacks that achieved beginning of symptom relief within 12 h did so with a single dose of sebetralstat (laryngeal: 96.0%; abdominal: 95.8%). Conventional on‐demand treatment was administered within 12 h for 3 (9.4%) laryngeal and 43 (8.1%) abdominal attacks.

**Conclusion:**

Oral sebetralstat enabled rapid treatment of laryngeal and abdominal attacks of all severities, was well tolerated, and provided early symptom relief.

## Graphical abstract

Mucosal hereditary angioedema attacks may progress rapidly and often lead to substantial morbidity. In the ongoing, international, 2‐year KONFIDENT‐S open‐label extension study, sebetralstat enabled early treatment and was generally well‐tolerated and effective in treating both laryngeal and abdominal HAE attacks.

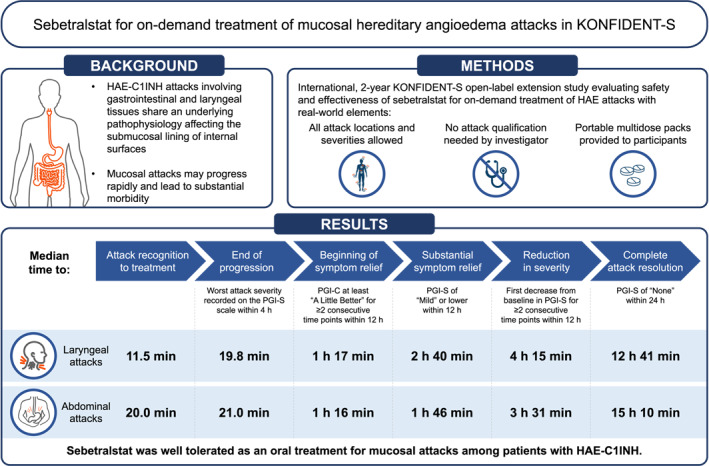

## 1 Introduction

People living with hereditary angioedema with C1 inhibitor deficiency (HAE‐C1INH), a genetic disorder that arises from the deficiency or dysfunction of C1 inhibitor, experience unpredictable and often debilitating attacks of tissue swelling [1, 2, 3]. Despite involving different anatomical locations, mucosal attacks, including those affecting the gastrointestinal tract and larynx, exhibit a common underlying pathophysiology and are prone to rapid progression [4, 5, 6].

Gastrointestinal attacks lead to severe pain, nausea, vomiting, and in some cases, temporary obstruction due to the thickening of the bowel wall [6]. In some severe abdominal attacks, circulatory shock occurs secondary to hypovolemia [6]. In the larynx, even minor edema can rapidly progress to life‐threatening airway compromise. Although laryngeal attacks represent roughly 1%–3% of all swelling attacks, over 50% of people living with HAE will experience these attacks at least once in their lifetime [3, 7, 8, 9]. The risk of mortality from untreated laryngeal attacks persists despite the availability of efficacious on‐demand treatments [3, 5, 10]. In a previously reported case series, death occurred within 20 min in 4 of 36 (11.1%) laryngeal attacks, with the attack becoming fatal prior to the administration of on‐demand treatment in all cases [10]. Early treatment of laryngeal attacks has been shown to prevent the need for invasive procedures such as endotracheal intubation or tracheotomy [5].

Consistent with these findings, early HAE treatment guidelines that were written at a time when availability and access to on‐demand treatments were very limited, prioritized the treatment of mucosal attacks over the treatment of subcutaneous/peripheral attacks [11, 12]. With increasing availability of treatment options, global clinical guidelines evolved to recommend treating all attacks (regardless of location and severity) and strongly recommend the prompt treatment of laryngeal attacks to prevent symptom progression and mitigate the risk of asphyxiation [13]. Nevertheless, in a recent US survey conducted in people living with HAE‐C1INH, the mean time to treatment of attacks affecting the throat or tongue was 2.0 h, while 25.0% of the respondents reported waiting 5.0 h or more to treat these attacks [14].

The delay and/or withholding of potentially life‐saving treatment, at least in part, may be due to the fact that until recently, all approved on‐demand treatments of HAE‐C1INH attacks required parenteral administration [1, 2]. This introduces significant barriers to rapid treatment in an acute and unpredictable setting. These barriers include challenges with storing and carrying of medication and injection‐related supplies, withholding of treatment due to the fear of pain and injection site reactions, finding a discreet and hygienic location for parenteral administration, and technical difficulties with self‐administration [14, 15, 16]. Consistent with these reported barriers, people living with HAE‐C1INH have reported a preference for less invasive treatment options [17, 18]. An oral on‐demand therapy may help enable quicker initiation of attack treatment in people living with HAE‐C1INH, potentially halting attack progression and reducing or eliminating the need for emergency interventions.

The plasma kallikrein inhibitor sebetralstat is the first orally administered treatment approved by the US Food and Drug Administration as well as the UK Medicines & Healthcare Products Regulatory Agency for the on‐demand treatment of HAE attacks in adults and children aged ≥ 12 years [19, 20]. In the randomized, double‐blind, placebo‐controlled Phase 2 trial, abdominal attacks were eligible for treatment, but laryngeal attacks were excluded due to concerns of potentially treating laryngeal attacks with placebo [21]. In the subsequent pivotal, randomized, double‐blind, placebo‐controlled Phase 3 KONFIDENT (NCT05259917) trial, all attack locations and severity levels were eligible except for severe laryngeal attacks [22]. Across all attacks, sebetralstat demonstrated faster times to beginning of symptom relief, reduction in attack severity, and complete attack resolution compared with placebo and was well‐tolerated in participants with HAE‐C1INH [22]. However, due to the event‐driven trial design, the number of laryngeal attacks included in the analysis was limited and precluded analysis of this subgroup in KONFIDENT [22]. Following the peer‐reviewed publications of the Phase 2 and Phase 3 trial results [21, 22], editorials raised questions as to whether the swelling associated with oropharyngeal or gastrointestinal attacks could impair the effectiveness of orally administered on‐demand treatments either due to difficulty swallowing or reduced intestinal absorption [23, 24].

The long‐term safety and effectiveness of sebetralstat is being studied in the ongoing 2‐year KONFIDENT‐S open‐label extension (OLE; NCT05505916; EudraCT: 2021‐001176‐42). The first prespecified interim analysis, which included treatment of 640 attacks, demonstrated that sebetralstat enabled compliance with treatment guidelines and generally corroborated the safety and efficacy profile observed in the KONFIDENT Phase 3 trial [25]. A total of 257 mucosal attacks were evaluated, including 193 involving the abdomen only and 14 involving the larynx. This second prespecified interim analysis of KONFIDENT‐S added more than 1000 additional attacks treated with sebetralstat, including a much larger number of mucosal attacks. This allows for a more comprehensive assessment of the safety and effectiveness of sebetralstat, specifically as an oral on‐demand treatment for mucosal HAE attacks, which are associated with substantial morbidity. This investigation is crucial to address the specific concerns about oral administration in this clinical context and to provide critical evidence supporting the use of a more patient‐preferred treatment option that may overcome the barriers associated with injectable therapies.

## 2 Materials and Methods

### 2.1 Study Design

The study design and eligibility criteria of the KONFIDENT‐S OLE have been previously described [25]. Participants were enrolled after completing KONFIDENT, or if they were ≥ 12 years of age with a confirmed diagnosis of HAE‐C1INH (Type 1 or Type 2) and ≥ 2 documented HAE attacks within 3 months of enrollment. Participants receiving long‐term prophylaxis (LTP) must have been on a stable dose and regimen for at least 3 months before screening and throughout the duration of the study. Consistent with global treatment guidelines [1, 2], participants were instructed to self‐administer on‐demand treatment (sebetralstat 600 mg film‐coated tablets) as early as possible for each attack. An optional second administration of sebetralstat was permitted after at least 3 h following the first administration but was limited to 2 administrations within 24 h (i.e., maximum 1200 mg total daily dose). Participants were permitted to administer conventional on‐demand treatment as per their usual treatment regimen after sebetralstat administration, provided they deemed their HAE attack symptoms severe enough. Participants were instructed to treat laryngeal attacks immediately with conventional on‐demand therapy if the attack symptoms worsened after the initial sebetralstat administration. This prespecified interim analysis includes attacks that participants reported as affecting the larynx/throat with or without involvement of other attack locations (i.e., laryngeal attacks) or the abdomen only (i.e., abdominal attacks).

### 2.2 Study Endpoints

Safety was assessed primarily by adverse event reporting and supplemented by laboratory tests and vital sign measurements. Effectiveness assessments included (1) time to beginning of symptom relief, defined as a rating of at least “A Little Better” on the Patient Global Impression of Change (PGI‐C) scale for at least 2 consecutive time points within 12 h of the first dose of sebetralstat; (2) time to first incidence of decrease from baseline in Patient Global Impression of Severity (PGI‐S) rating for at least 2 consecutive time points within 12 h of the first dose of sebetralstat; and (3) time to complete attack resolution, defined as a PGI‐S rating of “None” within 24 h of the first dose of sebetralstat. Post hoc analyses assessed (1) time to end of attack progression, defined as the time at which the worst attack severity was recorded on the PGI‐S scale within 4 h of treatment administration of the first dose of sebetralstat and (2) time to substantial symptom relief, defined as a PGI‐S rating of “Mild” or lower within 12 h of the first dose of sebetralstat (for attacks that had progressed to moderate severity or worse at baseline).

Participants recorded information related to sebetralstat‐treated attacks in an electronic diary (attack location, attack symptoms, date and time of onset, attack severity, time of sebetralstat administration(s), and use of conventional on‐demand treatment) at defined intervals (every 0.5 h for the first 2 h, every hour from 2 to 4 h, every 2 h from 4 to 8 h, and at 12 h, 18 h, and 24 h).

### 2.3 Statistics

Safety and effectiveness were analyzed in a subset of patients who had experienced at least 1 laryngeal or abdominal attack at the time of clinical data cutoff. Data were summarized descriptively; no inferential statistical analyses were performed. Continuous data are presented using descriptive statistics (i.e., *n*, mean, standard deviation, median, first and third quartiles, minimum, and maximum). Categorical data are presented using the participant count and percentage in each category. For the summary statistics of all numerical variables (unless otherwise specified), minimum and maximum are displayed to the same level of precision as the data collected. Attacks were treated as right‐censored at 0 h if a time‐to‐event result could not be derived. Conventional treatment administration was censored to the end of the analysis window.

## 3 Results

### 3.1 Participants

Between October 21, 2022 and September 14, 2024 (data cutoff), 134 participants enrolled from 72 sites in 23 countries administered sebetralstat for at least one attack in KONFIDENT‐S. Of the 134 participants, 16 participants (11.9%) experienced attacks involving the larynx and 102 participants (76.1%) experienced abdominal attacks that were treated with sebetralstat (Table 1). Participants experiencing laryngeal and abdominal attacks were predominantly female (62.5% and 69.6%, respectively), with a median (range) age of 44.5 years (15.0–67.0) for laryngeal attacks and 36.0 years (12.0–77.0) for abdominal attacks. Adolescents (12 to < 18 years of age) accounted for 12.5% and 16.7% of these groups, respectively.

**TABLE 1:** Demographics and baseline characteristics.

	Participants experiencing laryngeal attacks^a^ (*n* = 16)	Participants experiencing abdominal attacks^b^ (*n* = 102)
Age, median (range), years	44.5 (15.0–67.0)	36.0 (12.0–77.0)
Age group, *n* (%)		
≥ 12 to < 18 years	2 (12.5)	17 (16.7)
≥ 18 to < 65 years	13 (81.3)	81 (79.4)
≥ 65 years	1 (6.3)	4 (3.9)
Sex, *n* (%)		
Female	10 (62.5)	71 (69.6)
Male	6 (37.5)	31 (30.4)
Race, *n* (%)		
Asian	2 (12.5)	13 (12.7)
Not reported	1 (6.3)	7 (6.9)
Other	0	6 (5.9)
White	13 (81.2)	76 (74.5)
BMI, median (IQR), kg/m^2^	29.6 (26.4–32.2)	24.7 (22.1–29.9)
HAE‐C1INH type, *n* (%)		
Type 1	16 (100)	95 (93.1)
Type 2	0	7 (6.9)
Current treatment regimen, *n* (%)		
On‐demand only	9 (56.3)	77 (75.5)
On‐demand + LTP	7 (43.8)	25 (24.5)
Targeted kallikrein‐inhibiting agent^c^	6 (85.7)	21 (84.0)
C1INH	1 (14.3)	4 (16.0)

Abbreviations: BMI, body mass index; HAE‐C1INH, hereditary angioedema with C1‐inhibitor deficiency; IQR, interquartile range; LTP, long‐term prophylaxis; *n*, number of participants.

^a^ Attacks that participants reported as affecting the larynx/throat with or without involvement of other attack locations.

^b^ Attacks involving the abdomen only.

^c^ Lanadelumab or berotralstat.

### 3.2 Attack Characteristics

The full analysis set included 1706 attacks treated with sebetralstat; 32 (1.9%) of these attacks involved the larynx and 533 (31.2%) were abdominal attacks (Table 2). At the time of treatment with sebetralstat, 25.0% of the 32 laryngeal attacks were mild, 46.9% were moderate, and 28.1% were severe or very severe, while 29.6% of the 533 abdominal attacks were mild, 42.6% were moderate, and 27.8% were severe or very severe (as reported by the participant). The median (IQR) time from attack onset to treatment with sebetralstat was 11.5 min (1.0–34.0) and 20.0 min (1.0–61.0) in participants treating laryngeal and abdominal attacks, respectively.

**TABLE 2:** Attack characteristics.

	Laryngeal attacks (*n* = 32)	Abdominal attacks (*n* = 533)
Attack location (participant self‐reported), *n* (%)^a^		
Abdomen (only)^b^	N/A	533 (100)
Larynx/throat (any)^c^	32 (100)	N/A
Baseline PGI‐S category, *n* (%)		
Mild^d^	8 (25.0)	158 (29.6)
Moderate	15 (46.9)	227 (42.6)
Severe/very severe	9 (28.1)	148 (27.8)

Abbreviations: IQR, interquartile range; *n*, number of participants; N/A, not applicable; PGI‐S, Patient Global Impression of Severity.

^a^ Participants with multiple attack locations are counted once in each reported location.

^b^ Abdominal attacks with simultaneous involvement of other attack locations were excluded from this analysis.

^c^ Additional involvement of head/face/neck: *n* (%) = 8 (25.0%), additional mucosal attack location (abdomen): *n* (%) = 2 (6.3%), additional subcutaneous attack location: arms/hands and legs/feet: *n* (%) = 1 (3.1%) each.

^d^ Includes attacks with PGI‐S rating of “None”: *n* (%) = 1 (3.1%) for laryngeal attacks and *n* (%) = 4 (0.8%) for abdominal attacks.

### 3.3 Safety and Tolerability

At data cutoff, 7 (43.8%) participants with laryngeal attacks experienced 14 treatment‐emergent adverse events (TEAEs) and 36 (35.3%) participants with abdominal attacks experienced 91 TEAEs (Tables 3 and 4). No participants reported difficulty swallowing sebetralstat during laryngeal or abdominal attacks. Treatment‐related TEAEs occurred in 1 (6.3%) participant experiencing a laryngeal attack. One instance each of Grade 2 nausea and Grade 2 vomiting occurred in the same participant who experienced a laryngeal and an abdominal attack; this participant discontinued the study due to these events. Additionally, treatment‐related TEAEs occurred in 6 (5.9%) participants experiencing an abdominal attack, which included Grade 2 flu‐like symptoms, cutaneous burning, diarrhea (3 events), headache, myalgia (bilateral arm and bilateral leg [1 event each]), and 2 events of Grade 1 vomiting (Table 3).

**TABLE 3:** Safety.

Participants, *n* (%)	Participants experiencing laryngeal attacks (*n* = 16)	Participants experiencing abdominal attacks (*n* = 102)
**Any TEAE**	**7 (43.8)**	**36 (35.3)**
Treatment‐related	1 (6.3)^a^	6 (5.9)^b^
**Serious TEAE** ^c^	**2 (12.5)** ^d^	**2 (2.0)** ^e^
Treatment‐related	0	0
**Severe TEAE** ^f^	**3 (18.8)**	**2 (2.0)**
Treatment‐related	0	0
**Any TEAE leading to permanent discontinuation**	**1 (6.3)** ^a^	**2 (2.0)** ^g^
**Any TEAE leading to death**	0	0

*Note:* Bold values indicate the treatment‐emergent adverse events. The first number is the absolute number and the number in parentheses is the percent of the population with laryngeal and abdominal attacks, respectively.

Abbreviations: *n*, number of participants; TEAE, treatment‐emergent adverse event.

^a^ Treatment‐related nausea and vomiting (grade 2) occurred in the same participant, who experienced an attack involving the larynx and abdomen. These events led to treatment discontinuation.

^b^ Treatment‐related flu‐like symptoms, cutaneous burning, diarrhea (3 events), headaches, myalgia (bilateral arm and bilateral leg [1 event each]; all grade 2), and vomiting (2 events, grade 1) occurred in 6 participants, who experienced an attack involving the abdomen only.

^c^ Serious TEAE was defined as any untoward medical occurrence that at any dose results in death, is life‐threatening, requires inpatient hospitalization or prolongation of existing hospitalization, results in persistent or significant disability/incapacity, is a congenital anomaly/birth defect, or is an important medical event by medical and scientific judgment.

^d^ Serious AEs resulting in hospitalization (but considered unrelated to treatment) were 1 event of grade 3 viral meningitis occurring in 1 participant and 2 events of laryngeal HAE attack occurring in 1 participant.

^e^ Serious AEs were 1 event of Waldenstrom's macroglobulinemia occurring in 1 participant (considered unrelated to treatment) and 1 event each of headache, hyperthermia, and transcranial mass occurring in 1 participant (considered unrelated to treatment).

^f^ Severe (grade 3 or 4) TEAEs were evaluated by investigators according to the Toxicity Grading Scale for Healthy Adult and Adolescent Volunteers Enrolled in Preventive Vaccine Clinical Trials.

^g^ TEAEs leading to treatment discontinuation were 1 event of intracranial expansive lesion (unrelated to treatment), and 1 event of nausea (grade 2, considered treatment‐related).

**TABLE 4:** Summary of on‐treatment TEAEs by system, organ, class and preferred term.

Participants, *n* (%)	Participants experiencing laryngeal attacks (*n* = 16)	Participants experiencing abdominal attacks (*n* = 102)
On‐treatment TEAEs^a^		
Blood and lymphatic system disorders		
Lymphadenopathy	1 (6.3)	0
Congenital, familial, and genetic disorders		
HAE attack	1 (6.3)	0
Gastrointestinal disorders		
Colitis ulcerative	1 (6.3)	0
Diarrhea	0	1 (1.0)
Nausea	1 (6.3)	1 (1.0)
Vomiting	1 (6.3)	3 (2.9)
General disorders and administration site conditions		
Influenza like illness	0	1 (1.0)
Infections and infestations		
Nasopharyngitis	0	4 (3.9)
Pharyngitis streptococcal	0	1 (1.0)
Vulvovaginal mycotic infection	0	1 (1.0)
Musculoskeletal and connective tissue disorders		
Back pain	0	1 (1.0)
Myalgia	0	1 (1.0)
Osteochondrosis	0	1 (1.0)
Nervous system disorders		
Headache	0	3 (2.9)
Psychiatric disorders		
Depression	0	1 (1.0)
Skin and subcutaneous tissue disorders		
Skin burning sensation	0	1 (1.0)
Urticaria	0	1 (1.0)

Abbreviations: HAE, hereditary angioedema; *n*, number of participants; TEAE, treatment‐emergent adverse event.

^a^ TEAEs associated to attacks with missing baseline primary location were excluded from analysis.

Two (12.5%) participants with laryngeal attacks experienced serious TEAEs that were not considered related to sebetralstat: 1 event of Grade 3 viral meningitis occurring in 1 adult participant and 2 events of “laryngeal HAE attack” occurring in 1 adolescent that resulted in precautionary hospitalization. Similarly, 2 (2.0%) participants with abdominal attacks experienced serious TEAEs that were not considered related to sebetralstat: 1 event of Waldenstrom's macroglobulinemia occurring in 1 adult participant and 1 event each of headache, hyperthermia, and transcranial mass occurring in 1 adult participant.

### 3.4 Effectiveness

**FIGURE 1:**
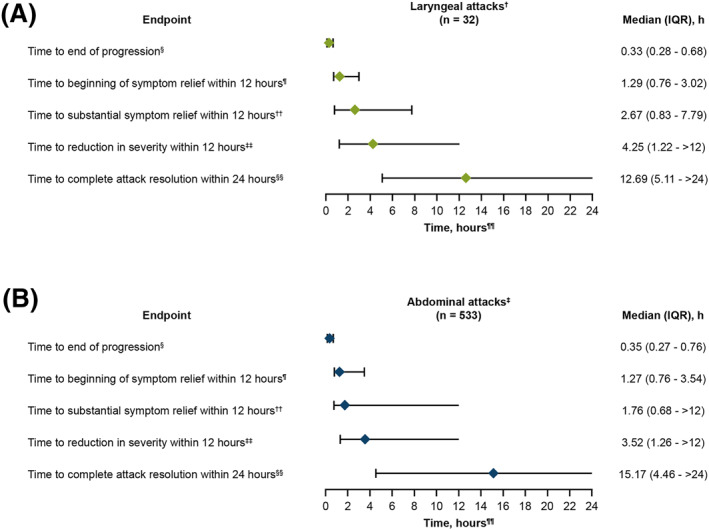
Effectiveness of sebetralstat in laryngeal (A) and abdominal attacks (B). ^†^Median attack frequency of 0.14 (IQR, 0.10–0.42) sebetralstat‐treated laryngeal attacks per month. ^‡^Median attack frequency of 0.44 (IQR, 0.25–0.83) sebetralstat‐treated abdominal attacks per month. ^§^Defined as the time at which the worst attack severity was recorded within 4 h on the PGI‐S scale. ^¶^Defined as a PGI‐C rating of at least “A Little Better” for at least 2 consecutive time points within 12 h, allowing for missing data entries. ^††^Defined as a PGI‐S rating of “Mild” or lower within 12 h of the first dose of sebetralstat. ^‡‡^Defined as time to first incidence of decrease from baseline in PGI‐S rating for at least 2 consecutive time points within 12 h of the first dose of sebetralstat. ^§§^Defined as a PGI‐S rating of “None” within 24 h of the first dose of sebetralstat. ^¶¶^Diamonds are the medians met within time window. Error bars display IQR.

Median (IQR) time to beginning of symptom relief (defined as a PGI‐C rating of at least “A Little Better” for at least 2 consecutive time points) within 12 h was 1.29 h (0.76–3.02) for laryngeal attacks and 1.27 h (0.76–3.54) in abdominal attacks (Figure 1). Median (IQR) time to reduction in attack severity within 12 h was 4.25 h (1.22 to > 12) for laryngeal attacks and 3.52 h (1.26 to > 12) for abdominal attacks, and median (IQR) time to complete attack resolution within 24 h was 12.69 h (5.11 to > 24) for laryngeal attacks and 15.17 h (4.46 to > 24) for abdominal attacks (Figure 1). In post hoc analyses, median (IQR) time to end of attack progression was 0.33 h (0.28–0.68) for laryngeal attacks and 0.35 h (0.27–0.76) for abdominal attacks; the median (IQR) time to substantial symptom relief within 12 h was 2.67 h (0.83–7.79) for laryngeal attacks and 1.76 h (0.68 to > 12) for abdominal attacks (Figure 1).

**FIGURE 2:**
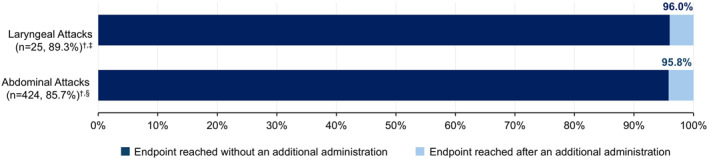
Proportion of attacks achieving beginning of symptom relief prior to or without an additional administration of sebetralstat. ^†^Proportion of attacks reaching the endpoint within 12 h. ^‡^Out of 28 evaluable laryngeal attacks with post‐baseline assessments. ^§^Out of 495 evaluable abdominal attacks with post‐baseline assessments.

Of the 32 laryngeal attacks, 4 (12.5%) were treated with a second administration of sebetralstat within 12 h and 3 (9.4%) were treated with conventional attack medication within 12 h; the time of conventional treatment administration was not recorded for 1 attack. For 2 attacks, conventional treatment was administered after a single administration of sebetralstat (in one case, conventional on‐demand treatment was administered 1 min after sebetralstat administration [PGI‐S at the time of treatment was rated as “Very Severe”]). For 1 attack, conventional treatment was administered after 2 administrations of sebetralstat, 11.7 h after the first administration of sebetralstat. Of the 533 abdominal attacks, 95 (17.8%) were treated with a second administration of sebetralstat within 12 h and 43 (8.1%) were treated with conventional attack medication within 12 h. Most of the attacks affecting the larynx (96.0%) and abdominal attacks (95.8%) that achieved beginning of symptom relief within 12 h did so with a single dose of sebetralstat (Figure 2).

Among participants who treated laryngeal (*n =* 16) or abdominal (*n =* 102) attacks with sebetralstat, 9 (56.3%) and 77 (75.5%) were on an on‐demand only treatment regimen, respectively, whereas 7 (43.8%) and 25 (24.5%) were on an on‐demand and LTP treatment regimen at baseline (Table 1). For laryngeal attacks, the median (IQR) time to beginning of symptom relief within 12 h was 1.27 h (0.52–2.49) in participants who used on‐demand treatment only and 1.36 h (0.94–4.09) in participants who received on‐demand and LTP treatment; corresponding times for abdominal attacks were 1.31 h (0.76–3.94) and 0.96 h (0.48–2.18), respectively. Median time to reduction in attack severity within 12 h was 2.43 h (0.80 to > 12) and 6.04 h (1.59 to > 12) for laryngeal attacks and 4.46 h (1.28 to > 12) and 1.78 h (0.81 to > 12) for abdominal attacks in the on‐demand only and in the on‐demand and LTP treatment subgroups, respectively. Median time to complete attack resolution within 24 h was 12.71 h (3.80 to > 24) and 12.67 h (9.02 to > 24) for laryngeal attacks and 20.27 h (5.03 to > 24) and 8.41 h (2.76–21.08) for abdominal attacks in the on‐demand only and in the on‐demand and LTP treatment subgroups, respectively.

## 4 Discussion

Hereditary angioedema (HAE‐C1INH) attacks, particularly those affecting the larynx and gastrointestinal tract, can be severe and life‐threatening [1, 2, 10]. Given the unpredictable nature and potentially rapid progression of these attacks, prompt and effective on‐demand treatment is crucial [10]. The data from KONFIDENT‐S provides valuable insights into the real‐world safety and effectiveness of sebetralstat, the first approved oral on‐demand treatment for HAE attacks in adults and children aged ≥ 12 years [19, 20], specifically in the context of mucosal attacks [19].

Consistent with the safety and effectiveness profile of sebetralstat across all attacks, our findings demonstrate that sebetralstat was generally well‐tolerated and effective in treating both laryngeal and abdominal HAE attacks. It is noteworthy that the overlap in clinical presentation between symptoms of abdominal attacks and gastrointestinal TEAEs can present a challenge in distinguishing between disease‐related symptoms and treatment‐emergent effects. Importantly, no participant reported difficulty swallowing sebetralstat during laryngeal or abdominal attacks, directly addressing a key concern raised in previous editorials regarding the potential impairment of orally administered treatments by mucosal swelling [23, 24]. This suggests that oral administration remains highly feasible during mucosal attacks, which is a significant advantage over parenteral treatment options that are associated with considerable barriers to rapid self‐administration [14].

Consistent with its relative ease of use and portability, sebetralstat enabled early treatment of both laryngeal and abdominal attacks, with a median (IQR) time to treatment of 11.5 min (1.0–34.0) and 20.0 min (1.0–61.0), respectively. This finding aligns with HAE treatment guidelines, which strongly recommend patients consider prompt treatment of all attacks, especially those involving the larynx [1, 2]. In comparison, the median time from laryngeal attack recognition to icatibant treatment was 2.0 h (IQR, 1.0–8.0) in the Icatibant Outcome Survey (IOS) [26] and 3.15 h (range, 0.85–94.50) with pdC1INH (Berinert) in the open‐label I.M.P.A.C.T.2 study [27]. In a post hoc analysis, 47.0% of laryngeal attacks were treated with pdC1INH (Cinryze) > 4.0 h after attack onset [28].

The median time to beginning of symptom relief was similar for both laryngeal (1.29 h) and abdominal (1.27 h) attacks. These times indicate a relatively rapid onset of action for sebetralstat across mucosal attack locations. The participant reporting 2 events of vomiting experienced beginning of symptom relief in 1.79 and 1.27 h, respectively, which is consistent with the efficacy observed across all abdominal attacks in this analysis. While an impact on efficacy cannot be fully excluded, these findings show no measurable effect in this case. The median time to end of attack progression was also comparable (median of 20.0 min for laryngeal and 21.0 min for abdominal attacks; Figure 1), suggesting that sebetralstat can effectively halt attack progression shortly after administration. Furthermore, a high proportion of both laryngeal (96.0%) and abdominal (95.8%) attacks that achieved time to beginning of symptom relief within 12 h did so with a single 600 mg dose of sebetralstat, underscoring its efficacy in this setting.

Comparisons of effectiveness to injectable on‐demand treatments are limited by differences in study populations, attack eligibility requirements (e.g., minimum severity), assessment scales, and endpoint definitions [13]. Only studies evaluating intravenous rhC1INH used a comparable efficacy endpoint to measure time to beginning of symptom relief as assessed in KONFIDENT‐S [29]. In a pooled analysis of randomized controlled studies and open label‐extensions evaluating rhC1INH (NCT00225147, NCT01188564), the median time to beginning of symptom relief was approximately 1.1 h for oro‐facial‐pharyngeal‐laryngeal attacks and approximately 1.0 h for abdominal attacks treated with rhC1INH, which were generally similar to the median times observed in KONFIDENT‐S [30]. Notably, a severity of ≥ 50 mm on the VAS with no improvement in attack symptoms for 1.0 h prior to dosing (i.e., ≥ 20 mm reduction in VAS score), was required for an attack to qualify for rhC1INH treatment [5, 30], whereas no minimum severity (assessed by PGI‐S) was required in KONFIDENT‐S. Higher pre‐treatment severity for rhC1INH may have made treatment effects easier to detect.

In this analysis, 12.5% of laryngeal attacks and 17.8% of abdominal attacks were treated with a second administration of sebetralstat within 12 h, which was similar to 12.1% and 13.7% of laryngeal and abdominal attacks treated with a second or third dose of icatibant in the IOS [26, 31]. This was also consistent with an analysis of pooled data from 4 clinical studies for ecallantide, a subcutaneous plasma kallikrein inhibitor approved in the United States only, in which 22 of 220 (10.0%) laryngeal attacks and 9.5% of abdominal attacks were treated with a second dose [32, 33]. It is more difficult to compare the rates of conventional treatment (rescue treatment) use across therapies as practice patterns (e.g., clinical setting) and study designs (e.g., use of the same treatment for rescue) differ substantially. However, it is noteworthy that participants in KONFIDENT‐S administered conventional on‐demand treatment in only a small percentage of laryngeal (9.4%) and abdominal (8.1%) attacks after sebetralstat, further supporting its effectiveness.

A limitation of this study is its open‐label design, which may introduce bias as there is no control group or active comparator. Limitations of this analysis include participants self‐reporting attack locations (i.e., without clinical confirmation by laryngoscopy or endoscopy). However, this approach reflects a real‐world setting, in which people living with HAE generally need to make treatment decisions without healthcare provider oversight. While this open‐label extension provided a larger dataset for mucosal attacks compared to the pivotal trials, the total number of attacks involving the larynx remained relatively small but consistent with what would be expected based on available epidemiologic data in HAE. Future studies with larger numbers of laryngeal attacks could further solidify these findings.

## 5 Conclusions

The KONFIDENT‐S OLE demonstrates that sebetralstat is a safe and effective oral on‐demand treatment for both laryngeal and abdominal HAE‐C1INH attacks, resulting in rapid onset of symptom relief. The ability to administer oral therapy, even during mucosal swelling, addresses a critical unmet need in HAE management and has the potential to significantly improve patient outcomes by enabling earlier intervention.

## Author Contributions

**Jonathan A. Bernstein:** writing – review and editing, conceptualization, writing – original draft, investigation, methodology. **Emel Aygören‐Pürsün:** writing – original draft, writing – review and editing, methodology, investigation, conceptualization. **Mauro Cancian:** writing – original draft, writing – review and editing, investigation. **Danny M. Cohn:** conceptualization, writing – original draft, writing – review and editing, methodology, investigation. **Timothy Craig:** writing – original draft, writing – review and editing, investigation. **Vesna Grivcheva‐Panovska:** writing – original draft, writing – review and editing, investigation. **Anthony Jordan:** writing – original draft, writing – review and editing, investigation. **William R. Lumry:** writing – original draft, writing – review and editing, investigation, conceptualization, methodology. **Inmaculada Martinez‐Saguer:** writing – original draft, writing – review and editing, investigation. **Isaac Melamed:** writing – original draft, investigation, writing – review and editing. **Kazumasa Ohmura:** writing – original draft, writing – review and editing, investigation. **Jonny Peter:** writing – original draft, writing – review and editing, investigation. **Marc A. Riedl:** conceptualization, writing – original draft, writing – review and editing, methodology, investigation. **Daniel F. Soteres:** writing – original draft, writing – review and editing, investigation. **Petra Staubach:** writing – original draft, writing – review and editing, investigation. **Marcin Stobiecki:** writing – original draft, writing – review and editing, investigation. **Ya‐Hsiu Chuang:** writing – original draft, formal analysis, writing – review and editing, conceptualization, methodology. **Michael D. Smith:** conceptualization, writing – original draft, writing – review and editing, formal analysis, methodology. **Christopher M. Yea:** conceptualization, writing – original draft, writing – review and editing, formal analysis, methodology. **Paul K. Audhya:** conceptualization, writing – original draft, writing – review and editing, formal analysis, methodology. **Andrea Zanichelli:** conceptualization, writing – original draft, writing – review and editing, investigation, methodology. **Henriette Farkas:** conceptualization, writing – original draft, writing – review and editing, methodology, investigation.

## Funding

This study was funded by KalVista Pharmaceutical Inc.

## Conflicts of Interest

J.A.B has received grants and/or honoraria from KalVista Pharmaceuticals, BioCryst, BioMarin, CSL Behring, Intellia Therapeutics, Ionis Pharmaceuticals, Pharming, Pharvaris, and Takeda/Shire and serves as the immediate past president of the American Academy of Allergy, Asthma & Immunology (AAAAI). E.A.‐P. has received grants, consulting fees, and honoraria, and/or served on advisory boards for KalVista Pharmaceuticals, Astria, BioCryst, BioMarin Europe, Centogene, CSL Behring, Intellia Therapeutics, Otsuka, Pharming Technologies, Pharvaris, and Takeda/Shire. M.C. has received honoraria and/or meeting/travel support from KalVista Pharmaceuticals, BioCryst, CSL Behring, Otsuka, Pharvaris, and Takeda. D.M.C. has received consulting fees paid to the institution, honoraria paid to the institution, medical writing support, meeting/travel support, research support; has served on advisory boards from KalVista Pharmaceuticals, Astria, BioCryst, CSL Behring, Intellia Therapeutics, Ionis Pharmaceuticals, Otsuka, Pharvaris, and Takeda; and has had a leadership role in the HAEi Medical Advisory panel for Central Eastern Europe and Benelux. T.C. has received grants, consulting fees, honoraria, and/or served on advisory boards and/or data safety monitoring for KalVista Pharmaceuticals, CSL Behring, GSK, Astria, Takeda, BioMarin Pharmaceutical Inc., BioCryst, Pharming, Ionis Pharmaceuticals, Grifols, Pharvaris, ADARx Pharmaceuticals, and Intellia Therapeutics; serves as the director for ACARE International Hereditary Angioedema Center and Alpha‐1 Resource Center; and is a member of the Medical Advisory Board for the HAE‐A. V.G.‐P. has received grants, consulting fees, honoraria, medical writing, and/or meeting/travel support from KalVista Pharmaceuticals, Astria, BioCryst, CSL Behring, Pharming, and Takeda. A.J. has received consulting fees and meeting/travel support from GlaxoSmithKline, CSL Behring, Takeda, and Pharvaris. W.R.L. has received consulting fees, grants and/or research support from KalVista Pharmaceuticals, Astria, BioCryst, BioMarin, CSL Behring, Express Scripts/CVS, Fresenius Kabi, Intellia, Magellan, Optum, Pharming, Pharvaris, Shire/Takeda, Optinose, Grifols, AstraZeneca, Sanofi/Regeneron, GSK, Ionis Pharmaceuticals, and Teva and has board membership with US Hereditary Angioedema Association Medical Advisory Board and DFW Metroplex Allergy Society. I.M.‐S. has received grants, consulting fees, and meeting/travel support and/or served on advisory boards and/or data safety monitoring for KalVista Pharmaceuticals, Takeda, CSL Behring, Pharming, BioCryst, Octapharma, and Pharvaris. I.M. has nothing to disclose. K.O. has received consulting fees and honoraria from Takeda, CSL Behring, BioCryst. J.P. has received grants, honoraria, and meeting/travel support and/or served on advisory boards for KalVista Pharmaceuticals, Takeda, Astria, CSL Behring, Sanofi, HAE‐i, and Pharvaris. M.A.R. has received grants, consulting fees, and funding for clinical trials from KalVista Pharmaceuticals, BioCryst, BioMarin, CSL Behring, Ionis Pharmaceuticals, Pharvaris, Takeda, Astria, Celldex, Cycle Pharma, Grifols, Intellia Therapeutics, Pfizer, Pharming, and Sanofi/Regeneron. D.F.S. has received grants, consulting fees, and/or honoraria from KalVista Pharmaceuticals, BioCryst, BioMarin, CSL Behring, Pharming, Pharvaris, and Takeda. P.S.‐R. has received honoraria and/or meeting/travel support from KalVista Pharmaceuticals CSL Behring, Takeda, and BioCryst. M.S. has received honoraria and/or consulting fees from BioCryst, CSL Behring, and Takeda. Y.H.C., M.D.S., C.M.Y., and P.K.A. are employees and shareholders of KalVista Pharmaceuticals. A.Z. has received honoraria and meeting/travel support and/or served on advisory boards for KalVista Pharmaceuticals, BioCryst, CSL Behring, Pharvaris, and Takeda. H.F. has received grants paid to the institution, honoraria, medical writing support, and meeting/travel support; has served on advisory boards for KalVista Pharmaceuticals, Astria, BioCryst, CSL Behring, Intellia, Ono Pharmaceutical, Pharming, Pharvaris, and Takeda; and has had a leadership role on the Angioedema Centers of Reference and Excellence (ACARE) Steering Committee.

## Data Availability

The data that support the findings of this study are available on request from the corresponding author. The data are not publicly available due to privacy or ethical restrictions.

## References

[1] M. Maurer , M. Magerl , S. Betschel , et al., “The International WAO/EAACI Guideline for the Management of Hereditary Angioedema–The 2021 Revision and Update,” Allergy 77, no. 7 (2022): 1961–1990, 10.1111/all.15214. 35006617

[2] P. J. Busse , S. C. Christiansen , M. A. Riedl , et al., “US HAEA Medical Advisory Board 2020 Guidelines for the Management of Hereditary Angioedema,” Journal of Allergy and Clinical Immunology: In Practice 9, no. 1 (2021): 132–150.e3, 10.1016/j.jaip.2020.08.046. 32898710

[3] K. Bork , J. T. Anderson , T. Caballero , et al., “Assessment and Management of Disease Burden and Quality of Life in Patients With Hereditary Angioedema: A Consensus Report,” Allergy, Asthma and Clinical Immunology 17, no. 1 (2021): 40, 10.1186/s13223-021-00537-2. PMC8056543 33875020

[4] S. De Maat , Z. L. M. Hofman , and C. Maas , “Hereditary Angioedema: The Plasma Contact System Out of Control: Reply,” Journal of Thrombosis and Haemostasis 16, no. 11 (2018): 2349–2351, 10.1111/jth.14269. 30129108

[5] K. Bork , J. A. Bernstein , T. Machnig , and T. J. Craig , “Efficacy of Different Medical Therapies for the Treatment of Acute Laryngeal Attacks of Hereditary Angioedema Due to C1–Esterase Inhibitor Deficiency,” Journal of Emergency Medicine 50, no. 4 (2016): 567–580, 10.1016/j.jemermed.2015.11.008. 26826769

[6] K. Bork , P. Staubach , A. J. Eckardt , and J. Hardt , “Symptoms, Course, and Complications of Abdominal Attacks in Hereditary Angioedema Due to C1 Inhibitor Deficiency,” American Journal of Gastroenterology 101, no. 3 (2006): 619–627, 10.1111/j.1572-0241.2006.00492.x. 16464219

[7] A. Zanichelli , G. M. Azin , F. Cristina , R. Vacchini , and T. Caballero , “Safety, Effectiveness, and Impact on Quality of Life of Self‐Administration With Plasma‐Derived Nanofiltered C1 Inhibitor (Berinert^®^) in Patients With Hereditary Angioedema: The SABHA Study,” Orphanet Journal of Rare Diseases 13, no. 1 (2018): 51, 10.1186/s13023-018-0797-3. 29631595 PMC5891972

[8] A. Zanichelli , M. Mansi , G. M. Azin , et al., “Efficacy of On‐Demand Treatment in Reducing Morbidity in Patients With Hereditary Angioedema Due to C1 Inhibitor Deficiency,” Allergy 70, no. 12 (2015): 1553–1558, 10.1111/all.12731. 26304015

[9] K. Bork , J. Hardt , and G. Witzke , “Fatal Laryngeal Attacks and Mortality in Hereditary Angioedema Due to C1‐INH Deficiency,” Journal of Allergy and Clinical Immunology 130, no. 3 (2012): 692–697, 10.1016/j.jaci.2012.05.055. 22841766

[10] B. L. Zuraw , “Clinical Practice. Hereditary Angioedema,” New England Journal of Medicine 359, no. 10 (2008): 1027–1036, 10.1056/nejmcp0803977. 18768946

[11] T. Bowen , M. Cicardi , H. Farkas , et al., “Canadian 2003 International Consensus Algorithm for the Diagnosis, Therapy, and Management of Hereditary Angioedema,” Journal of Allergy and Clinical Immunology 114, no. 3 (2004): 629–637, 10.1016/j.jaci.2004.06.043. 15356569

[12] M. M. Gompels , R. J. Lock , M. Abinun , et al., “C1 Inhibitor Deficiency: Consensus Document,” Clinical and Experimental Immunology 139, no. 3 (2005): 379–394, 10.1111/j.1365-2249.2005.02726.x. 15730382 PMC1809312

[13] D. M. Cohn , D. F. Soteres , T. J. Craig , et al., “Interplay Between On‐Demand Treatment Trials for Hereditary Angioedema and Treatment Guidelines,” Journal of Allergy and Clinical Immunology 155, no. 3 (2025): 726–739, 10.1016/j.jaci.2024.12.1079. 39724968

[14] S. Christiansen , M. O'Connor , T. Craig , et al., “On‐Demand Treatment of Hereditary Angioedema Attacks: Patient‐Reported Utilization, Barriers, and Outcomes,” Annals of Allergy, Asthma, & Immunology 134, no. 5 (2025): 570–579, 10.1016/j.anai.2024.12.012. 39694088

[15] J. Mendivil , R. Murphy , M. de la Cruz , et al., “Clinical Characteristics and Burden of Illness in Patients With Hereditary Angioedema: Findings From a Multinational Patient Survey,” Orphanet Journal of Rare Diseases 16, no. 1 (2021): 94, 10.1186/s13023-021-01717-4. 33602292 PMC7893968

[16] S. Beyaz , S. Demir , N. Oztop , B. Colakoglu , S. Buyukozturk , and A. Gelincik , “How Satisfactory Is On‐Demand Icatibant From the Patients' Perspective in Real Life?,” Allergy and Asthma Proceedings 43, no. 2 (2022): 148–154, 10.2500/aap.2022.43.210104. 35317892

[17] Center for Biologics Evaluation and Research UFDA . The Voice of the Patient: Hereditary Angioedema (2018), https://www.fda.gov/media/113509/download.

[18] A. Valerieva , T. Caballero , M. Magerl , J. P. Frade , P. K. Audhya , and T. Craig , “Advent of Oral Medications for the Treatment of Hereditary Angioedema,” Clinical and Translational Allergy 14, no. 9 (2024): e12391, 10.1002/clt2.12391. 39331535 PMC11431061

[19] KalVista Pharmaceuticals, Inc . EKTERLY (Sebetralstat) US Prescribing Information (2025), https://www.kalvista.com/ekterly‐us‐prescribing‐information.pdf.

[20] KalVista Pharmaceuticals Ltd . Ekterly 300 Mg Film‐Coated Tablets (2025), https://mhraproducts4853.blob.core.windows.net/docs/789f8ee002136e09f13a44d7c5f7c51c6f2e0f87.

[21] E. Aygören‐Pürsün , A. Zanichelli , D. M. Cohn , et al., “An Investigational Oral Plasma Kallikrein Inhibitor for On‐Demand Treatment of Hereditary Angioedema: A Two‐Part, Randomised, Double‐Blind, Placebo‐Controlled, Crossover Phase 2 Trial,” Lancet 401, no. 10375 (2023): 458–469, 10.1016/s0140-6736(22)02406-0. 36774155

[22] M. A. Riedl , H. Farkas , E. Aygören‐Pürsün , et al., “Oral Sebetralstat for On‐Demand Treatment of Hereditary Angioedema Attacks,” New England Journal of Medicine 391, no. 1 (2024): 32–43, 10.1056/nejmoa2314192. 38819658

[23] A. Reshef and A. S. Grumach , “New Medications to Mitigate Attacks of Hereditary Angioedema: Does One Size Fit All?,” Lancet 401, no. 10375 (2023): 413–415, 10.1016/s0140-6736(23)00267-2. 36774145

[24] R. Ameratunga and H. J. Longhurst , “New Therapies for Type 1 and Type 2 Hereditary Angioedema,” New England Journal of Medicine 391, no. 1 (2024): 79–81, 10.1056/nejme2405299. 38819650

[25] H. Farkas , J. Anderson , L. Bouillet , et al., “Long‐Term Safety and Effectiveness of Sebetralstat: Interim Analysis of KONFIDENT‐S Open‐Label Extension,” Journal of Allergy and Clinical Immunology: In Practice (2025): Published Online August 29, 10.1016/j.jaip.2025.08.020. 40886933

[26] H. J. Longhurst , W. Aberer , L. Bouillet , et al., “The Icatibant Outcome Survey: Treatment of Laryngeal Angioedema Attacks,” European Journal of Emergency Medicine 23, no. 3 (2016): 224–227, 10.1097/mej.0000000000000292. 27116379 PMC4892758

[27] T. J. Craig , A. K. Bewtra , S. L. Bahna , et al., “C1 Esterase Inhibitor Concentrate in 1085 Hereditary Angioedema Attacks–Final Results of the I.M.P.A.C.T.2 Study,” Allergy 66, no. 12 (2011): 1604–1611, 10.1111/j.1398-9995.2011.02702.x. 21884533

[28] M. A. Riedl , W. R. Lumry , H. H. Li , et al., “Nanofiltered C1 Esterase Inhibitor for Treatment of Laryngeal Attacks in Patients With Hereditary Angioedema,” American Journal of Rhinology & Allergy 27, no. 6 (2013): 517–521, 10.2500/ajra.2013.27.3973. 24274230

[29] M. A. Riedl , J. A. Bernstein , H. Li , et al., “Recombinant Human C1‐Esterase Inhibitor Relieves Symptoms of Hereditary Angioedema Attacks: Phase 3, Randomized, Placebo‐Controlled Trial,” Annals of Allergy, Asthma, & Immunology 112, no. 2 (2014): 163–169.e1, 10.1016/j.anai.2013.12.004. 24468257

[30] J. W. Baker , J. A. Bernstein , J. R. Harper , A. Relan , and M. A. Riedl , “Efficacy of Recombinant Human C1 Esterase Inhibitor Across Anatomic Locations in Acute Hereditary Angioedema Attacks,” Allergy and Asthma Proceedings 39, no. 5 (2018): 359–364, 10.2500/aap.2018.39.4151. 29954477

[31] H. J. Longhurst , W. Aberer , L. Bouillet , et al., “Analysis of Characteristics Associated With Reinjection of Icatibant: Results From the Icatibant Outcome Survey,” Allergy and Asthma Proceedings 36, no. 5 (2015): 399–406, 10.2500/aap.2015.36.3892. 26314822

[32] A. L. Sheffer , A. J. MacGinnitie , M. Campion , L. E. Stolz , and W. E. Pullman , “Outcomes After Ecallantide Treatment of Laryngeal Hereditary Angioedema Attacks,” Annals of Allergy, Asthma, & Immunology 110, no. 3 (2013): 184–188.e2, 10.1016/j.anai.2012.12.007. 23548529

[33] H. H. Li , M. Campion , T. J. Craig , et al., “Analysis of Hereditary Angioedema Attacks Requiring a Second Dose of Ecallantide,” Annals of Allergy, Asthma, & Immunology 110, no. 3 (2013): 168–172, 10.1016/j.anai.2012.12.004. 23548526

